# Pity ’Tis ’Tis True

**Published:** 1889-01

**Authors:** 


### "PITY 'TIS, 'TIS TRUE."

The one thing that must not be investigated, must not be spoken of, must not be introduced into a college or into literature, is the grand pre-eminent fact—the modern Epiphany—the rolling away of the tombstone that hides our resurrection. The coming to earth and to human recognition of the angel hosts who have ever been looking down in love, but from whom benighted mortals in their darkness and *tabooing* ignorance have turned away and refused to hear the glorious message, the gospel of eternal life.

This grand epoch-making truth, which opens to mortal man a nobler destiny on earth as well as in heaven, is the sweetest, the noblest, the most inspiring and eloquent revelation that has ever been made since the earth became habitable by man.—BUCHANAN'S *Journal of Man.*

